# Epigenetic Alterations at Genomic Loci Modified by Gene Targeting in *Arabidopsis thaliana*

**DOI:** 10.1371/journal.pone.0085383

**Published:** 2013-12-26

**Authors:** Michal Lieberman-Lazarovich, Cathy Melamed-Bessudo, Sylvia de Pater, Avraham A. Levy

**Affiliations:** 1 Department of Plant Biology, University of Geneva, Geneva, Switzerland; 2 Department of Plant Sciences, The Weizmann Institute of Science, Rehovot, Israel; 3 Department of Molecular and Developmental Genetics, Institute of Biology, Leiden University, Leiden, The Netherlands; University of Georgia, United States of America

## Abstract

Gene Targeting (GT) is the integration of an introduced vector into a specific chromosomal site, via homologous recombination. It is considered an effective tool for precise genome editing, with far-reaching implications in biological research and biotechnology, and is widely used in mice, with the potential of becoming routine in many species. Nevertheless, the epigenetic status of the targeted allele remains largely unexplored. Using GT-modified lines of the model plant *Arabidopsis thaliana*, we show that the DNA methylation profile of the targeted locus is changed following GT. This effect is non-directional as methylation can be either completely lost, maintained with minor alterations or show instability in the generations subsequent to GT. As DNA methylation is known to be involved in several cellular processes, GT-related alterations may result in unexpected or even unnoticed perturbations. Our analysis shows that GT may be used as a new tool for generating epialleles, for example, to study the role of gene body methylation. In addition, the analysis of DNA methylation at the targeted locus may be utilized to investigate the mechanism of GT, many aspects of which are still unknown.

## Introduction

The ability to modify genomes in a precise manner provides a powerful tool for both basic and applied research [1,2]. It can be used to study gene function, to perform gene therapy or to improve crop plants. Several methods have been developed to allow for precise modifications of genomes. These include gene targeting (GT), DNA cleavage and mutagenesis by custom-designed endonucleases, site-specific recombination (e.g. Cre-Lox recombination), and oligo-mediated mutagenesis. These techniques enable targeted modifications, such as gene knock-outs, gene replacement, mutations or insertions and are routinely implemented in model organisms such as yeast [3] and mice [4]. 

**Figure 1:**
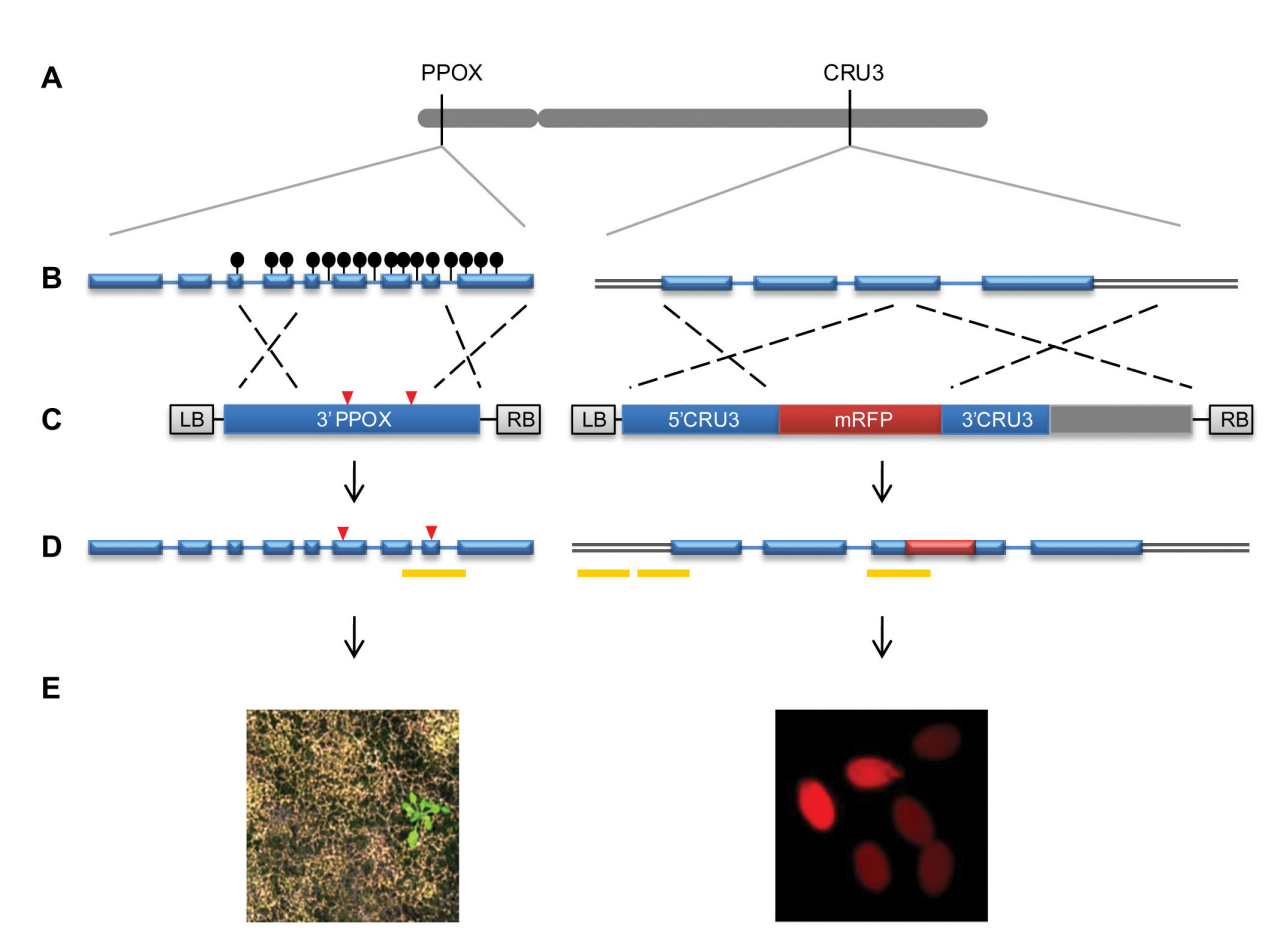
Genomic targets and gene targeting (GT) assays. (**A**) The *PPOX* and *CRUCIFERIN3* target loci are both located on chromosome 4 (in grey). (**B**) The target gene models with methylated cytosines, are shown as black lollipops. (**C**) The GT vectors are shown with indication of the left and right borders (LB and RB, respectively) of the *Agrobacterium tumefaciens* vector; red triangles indicate the two point mutations that confer herbicide resistance; mRFP (monomeric red fluorescence protein) was used as positive targeting marker; dashed lines indicate recombination between homologous genomic sequences. (**D**) The projected structure of the targeted loci following precise recombination with the targeting vectors is shown; Yellow bars correspond to the regions analyzed by bisulfite-sequencing. Blue rectangles, blue lines and double grey lines represent exons, introns and adjacent genomic regions, respectively. (**E**) *PPOX*-targeted plants are resistant to Butafenacil treatment (left) and *CRUCIFERIN3*-targeted seeds show red-fluorescence (right).

It was shown, for example, that the prolonged clotting times in a mouse model of haemophilia B can be corrected by in vivo GT [5], raising the possibility of using this strategy for the treatment of genetic disease. Another study demonstrated an efficient GT procedure in Nannochloropsis sp., a fast-growing, unicellular algae capable of accumulating large amounts of oil [6]. In higher plants, the low frequency of site-directed homologous recombination hindered GT from becoming routine. The frequency of GT events detected in early studies was in the range of 10^-3^ to 10^-6^ [7]. The finding that DNA double-strand breaks (DSBs) induce homologous-recombination-driven repair in plants [8] was exploited for enhancing GT by 2-3 orders of magnitude, using custom-designed nucleases such as Zinc Finger Nucleases (ZFNs) [9,10] Transcription Activator-Like Effector Nucleases (TALENs) [11] and the recently Clustered, Regularly Interspaced, Short Palindromic Repeats (CRISPR)-associated protein (Cas)[12]. It is therefore possible that GT will also become routine in plants in the near future, facilitating efforts to deal with challenges such as food and fuel security. Regardless of the technology employed, precise engineering of genomes, via GT, is assumed to enable controlled expression of the introduced gene as a result of its insertion into the original genomic context. However, the introduced DNA is devoid of the epigenetic modifications which have been established as critical in determining chromatin structure and gene expression patterns [13-16]. Thus, GT might bear an impact on the epigenetic status of the targeted allele, a hypothesis which was largely unexplored. To test this, we assessed the effect of GT on the pattern of DNA cytosine methylation, by analyzing two endogenous Arabidopsis target loci, both located on chromosome 4. The two loci differ in their methylation landscape, thus offering a contrasting perspective. The *PPOX* gene (At4g01690) is naturally enriched in CG gene-body methylation, in a region that encompasses one of the two bases to be modified by the targeting vector (Figure 1). In contrast, the *CRUCIFERIN3* gene (At4g28520) fully lacks cytosine methylation in the WT background [17,18] (Figure 1). Using these two genomic targets, we could determine whether there is a change, of hypo- or hypermethylation at the targeted allele.

## Materials and Methods

For the GT experiments, *Arabidopsis thaliana* plants (ecotype *Columbia*) were grown in controlled chambers at 21°C, with 16/8 hours of light/dark, respectively. The GT vector was transformed into inflorescences using the floral-dip method. Mature seeds were collected and screened for successful GT events. Genetic characterization of the GT lines was performed by PCR and Southern-blot assays. Methylation analysis of targeted lines was performed using the EpiTect kit (QIAGEN) for bisulfite conversion [19] of genomic DNA, according to the manufacturer's instructions with one modification: the number of incubation cycles was increased in order to ensure full conversion. Genomic DNA was extracted with the DNeasy Plant Mini Kit (QIAGEN), according to the manufacturer's protocol. Converted DNA was used for amplification of the target and PCR products were cloned and Sanger-sequenced. The number of clones that were sequenced in each experiment is indicated in the relevant supplementary table. For the *PPOX* locus analysis, pools of 4-8 young Arabidopsis plantlets were used in each sample. For the *CRUCIFERIN3* and *PROHIBITIN1* methylation analyses mature plant tissue were sampled, by pooling 2-6 plants from each line. Conversion rate was estimated at 99 to100%, by sequencing a fragment of the non-methylated PHAVOLUTA gene (At1g30490). Sequence analysis for calculating methylation fractions and for generating methylation plots was performed by the Kismeth software [20]. In order to independently determine the methylation patterns of the endogenous *PROHIBITIN1*and the copied *PROHIBITIN1*, we took an approach of size separation; the genomic DNA was digested with *Pvu*II, which generates a fragment of around 8300bp at the endogenous *PROHIBITIN1* locus, and a 4000bp fragment at the duplicated gene. Digested DNA was extracted from the gel in two separate reactions, corresponding to the 8300bp and 4000bp fragments, and then used for bisulfite conversion and sequencing.

## Results

**Figure 2:**
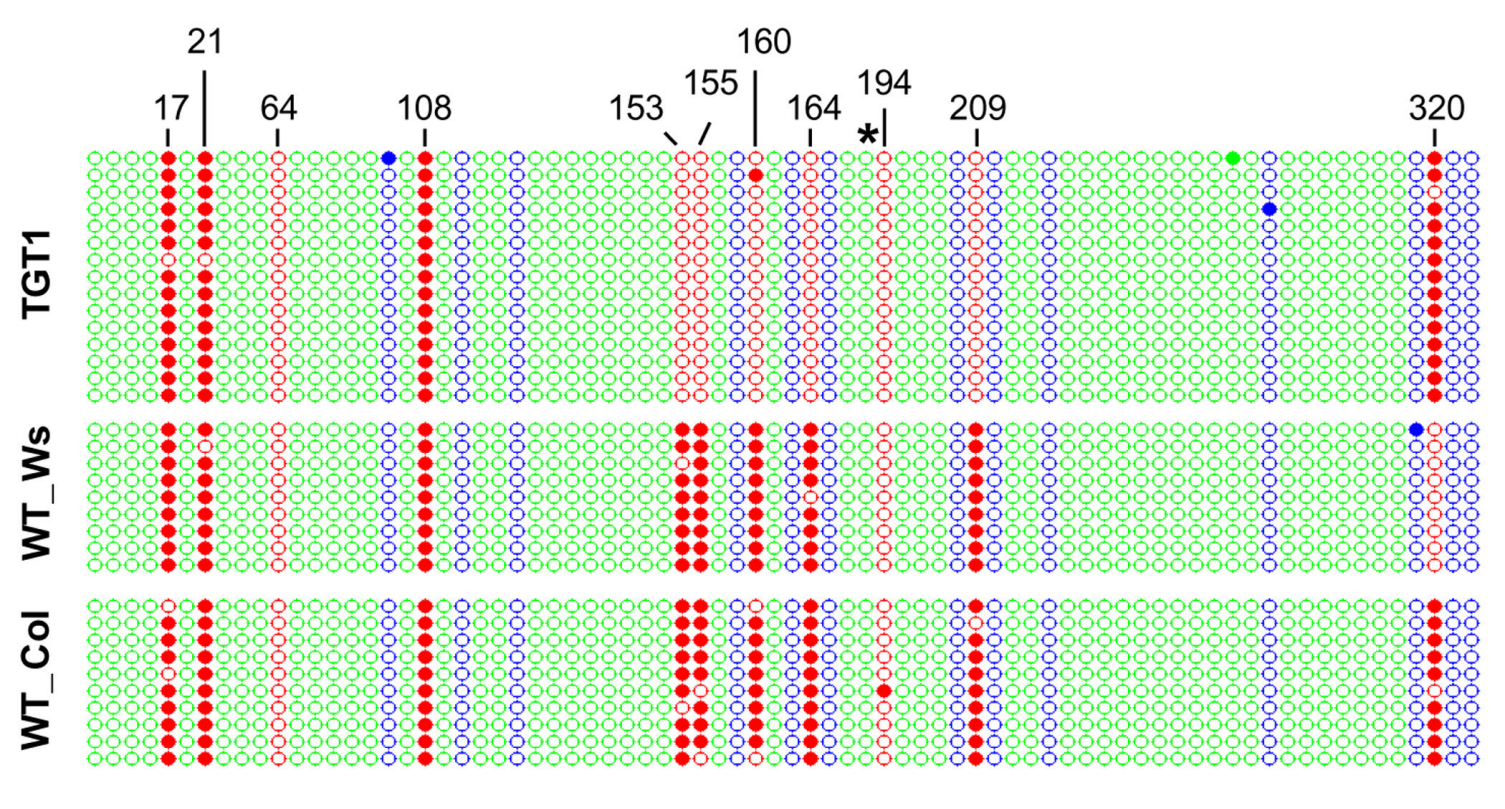
Loss of CG methylation at the targeted *PPOX* locus in the TGT-1 line. Cytosine methylation data obtained from bisulfite-sequencing of the PPOX fragment in the targeted line TGT-1 and two WT lines from ecotypes *Columbia (Col)* and *Wassilewskija* (Ws). Each circle represents a cytosine residue, either methylated (full circle) or non-methylated (empty circle). Cytosines are color-coded by their sequence context: red for CG, blue for CHG and green for CHH (H is C, T or A). Each row represents an independent clone. The numbers at the top of the columns indicate the position in base pairs, relative to the first base in this fragment. The second SNP introduced by the GT vector, is shown by an asterisk.

**Figure 3:**
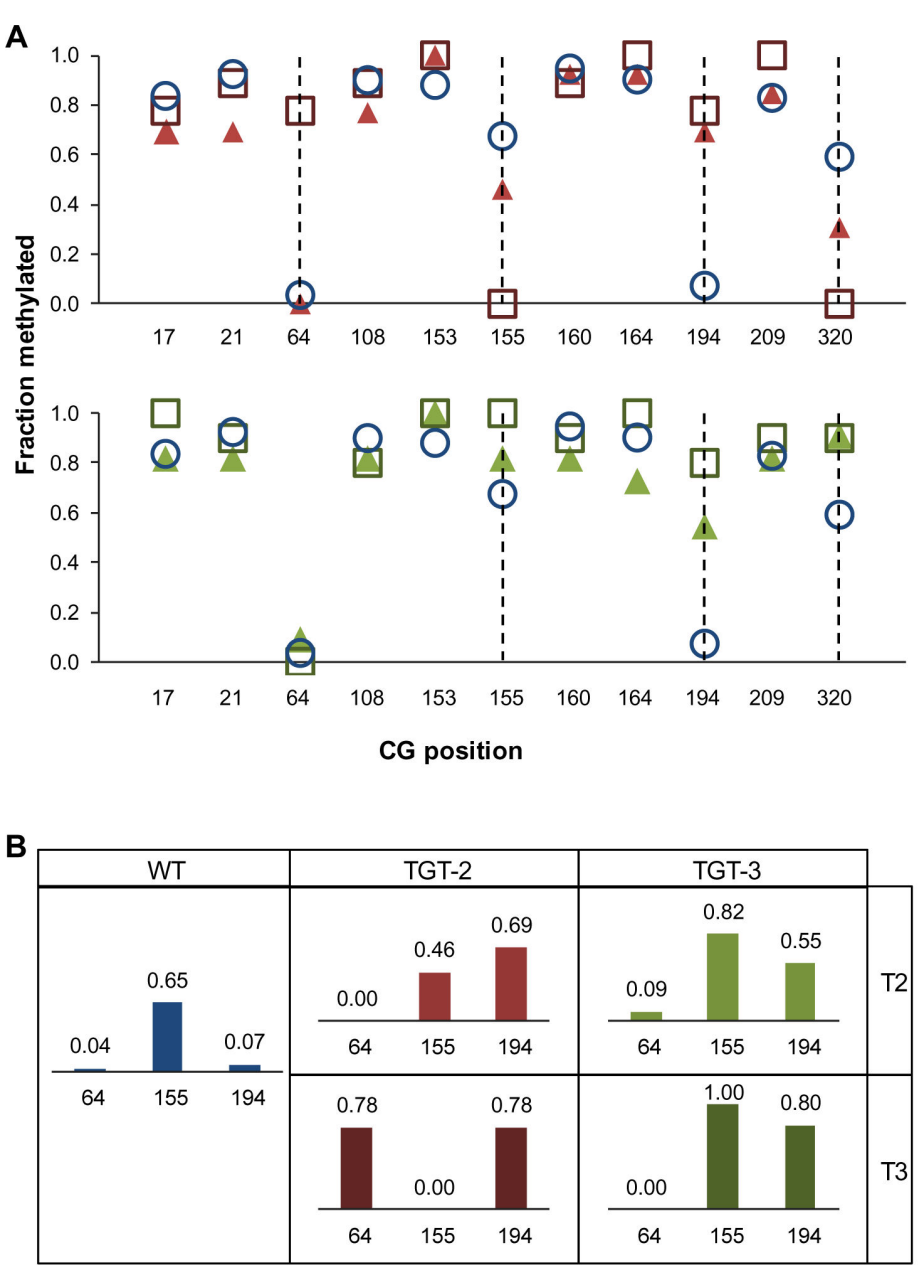
Methylation changes at the targeted *PPOX* locus in lines TGT-2 and TGT-3. (**A**) The methylation landscape in TGT-2 and TGT-3 lines (upper and lower graphs, respectively). Blue circles, triangles and squares correspond to WT, T2 and T3 generations, respectively. Dotted lines mark the CG positions at which the methylation level had changed relative to WT. (**B**) CG positions that lost methylation stability in the two targeted lines. CG position is denoted beneath each bar. The numbers above each bar denote the level of methylation as fraction.

**Figure 4:**
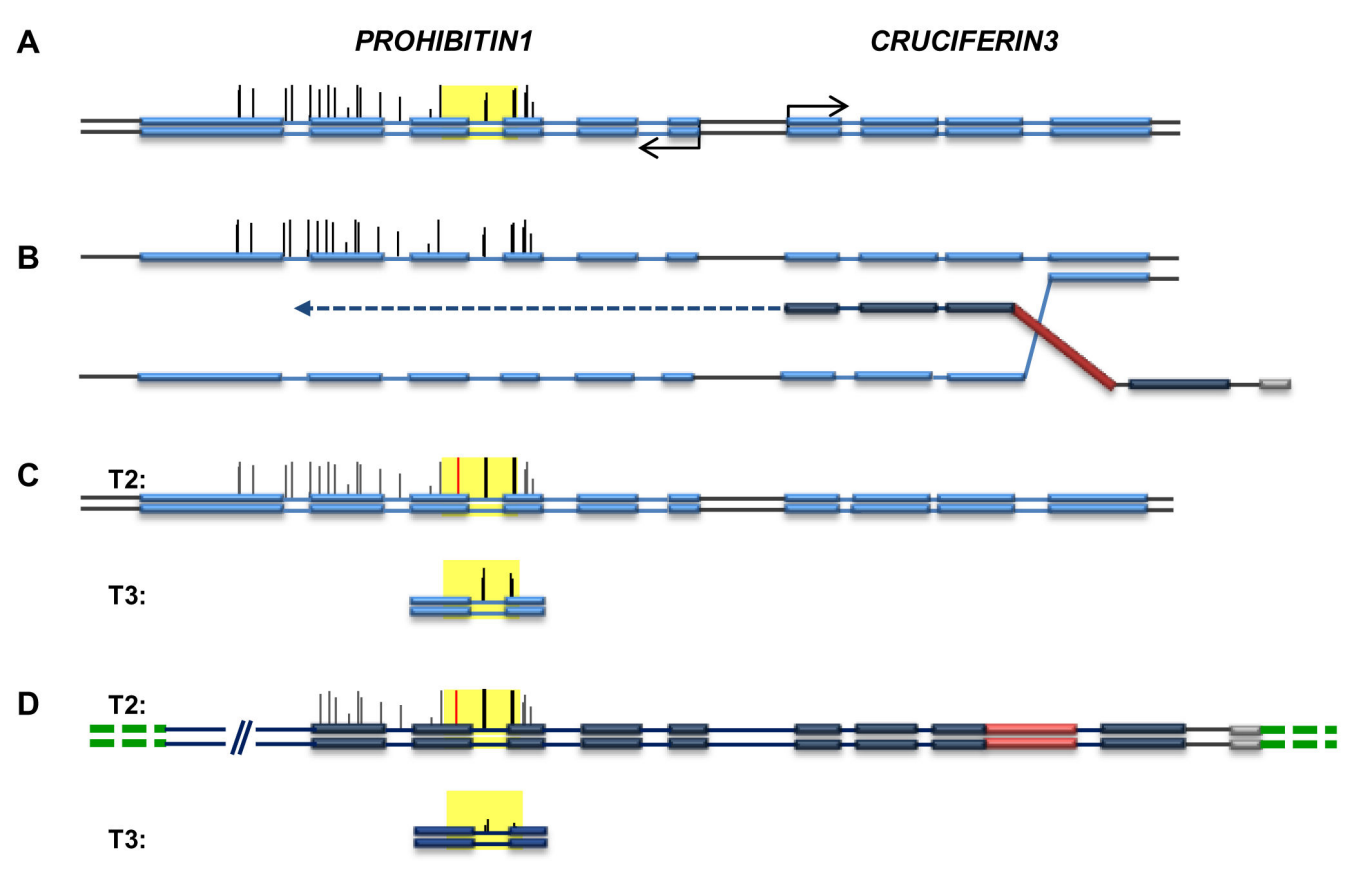
Ectopic Gene Targeting (EGT)-derived duplication of the *PROHIBITIN1* gene and subsequent changes in DNA methylation. (**A**) Schematic presentation of the WT *CRUCIFERIN3* locus, including the upstream *PROHIBITIN1* gene. Methylated cytosines are marked by vertical lines whose heights are proportional to the level of methylation (Cokus, Feng et al. 2008; Lister, O'Malley et al. 2008). The region analyzed by bisulfite-sequencing is highlighted in yellow. (**B**) The GT vector, containing sequences homologous to the target (dark blue) as well as the mRFP fluorescent marker (red), invades the *CRUCIFERIN3* target and primes DNA synthesis (dark blue dotted arrow), copying the *PROHIBITIN1* sequence. (**C**) and (**D**) Genetic and epigenetic consequences of EGT: (**C**) Methylation status at the endogenous copy, in T2 (upper scheme) and T3 (lower scheme) generations after GT. The red vertical line marks the newly methylated CG at position 18. (**D**) The vector including the *PROHIBITIN1* fragment that had integrated into chromosome 1 (green dotted lines) creating gene duplication. The methylation status of this duplicated copy is shown, in T2 (upper scheme) and T3 (lower scheme) generations after GT. Grey bars: the vector RB (right border) sequences.

Gene replacement of the endogenous *PPOX* locus, by a vector presenting one or two SNPs (Single Nucleotide Polymorphisms) that modify specific amino acids, confers herbicide resistance [21]. We analyzed three independent GT-derived lines by Southern blotting and sequencing, to confirm that gene targeting resulted in the precise replacement of the endogenous *PPOX* locus. These targeted lines did not contain randomly integrated copies of the vector in the genome (Figure S1), and were thus considered True Gene Targeting (TGT) events and consequently named TGT-1 [21], TGT-2, and TGT-3 (originally named C1 and T2, respectively [22]). Bisulfite-sequencing was performed on the CG-methylated region, encompassing the second SNP introduced by the GT vector (Figure 1), to enable comparison of the post-targeting methylation state of the targeted allele to that of the WT. In addition, methylation status in the progeny of the initial GT events was also evaluated. Two types of DNA methylation modifications were identified, that may have occurred at the targeted *PPOX* locus. The TGT-1 line, generated in the wild-type (WT) background (ecotype *Wassilewskija*, *Ws*), lost half of the total CG methylation in the examined region, reaching a level of 35.2%, compared to 69.7% in the wild-type *Ws* plant (Table S1). Methylation was virtually obliterated in the central region, encompassing six of the eleven CG sites as well as the second SNP introduced by the GT vector (Figure 2). This prominent pattern may possibly reflect the location and length of the conversion tract in the process of homology-mediated GT. The last CG site in this sequence, at position 320 (Figure 2), was methylated in TGT-1, but not in the WT (*Ws*). We found this specific site to be particularly unstable, as even in WT isolates of the same ecotype (*Columbia*), both full and lack of methylation was seen (Table S2). Since other CG sites in this region showed a consistent methylation pattern in different WT isolates, position 320 can be considered an outlier. To address the question of whether the observed loss of methylation has an influence on gene expression, we carried out expression analysis of the *PPOX* gene using two different primer pairs. As presented in Figure S2, the *PPOX* transcript level did not differ between TGT-1 and the WS line used as control. In contrast to the local depletion of methylation in the *PPOX* allele of the TGT-1 line, the overall methylation level was not dramatically changed in TGT-2 and TGT-3 [22], both in the *Columbia* background (Figure 3a). While the mean WT (*Columbia*) methylation level was 68.1%, the rates of CG methylation in the targeted allele of TGT-2 and TGT-3 were 66.4% and 74.4%, respectively (Table S1). Most CG sites (7 out of 11) presented methylation levels similar to WT, however, methylation at three sites (at positions 64, 155 and 194) was altered (Figure 3b) (Table S2). The CG dinucleotide at position 64 differed from the other two sites, presenting sharp increase in methylation only in the T3 generation of the TGT-2 line (Figure 3b). The CG sites at positions 155 and 194 exhibited altered methylation levels already in the T2 generation. Interestingly, this trend of change was further enhanced in the following generation (T3) (Figure 3b). At position 155, the WT methylation level was moderate (65%, Table S2). In the TGT-2 line, this position had lost some methylation in the first generation after GT, and completely lost all methylation in the T3 generation. In contrast, the TGT-3 line had gained methylation at this site, which rose to 90-100% in T3. In position 194, methylation was very low in WT (7%) but increased in the T2 generation of both TGT-2 and TGT-3 lines, and showed a further increase in T3, reaching methylation levels as high as 78-80% (Figure 3b). Non-CG methylation (i.e. CHG, CHH) was very low in this region (0 to 2.55% in WT, Table S1), a status that was maintained in the targeted lines, with the exception of the targeted allele of TGT-3, where CHG methylation was higher (Table S1). In order to confirm that changes in DNA methylation did not occur globally due to the transformation process, we have tested the methylation status of a non-target locus, which, similarly to the *PPOX* gene, presents CG body methylation in the WT background. As shown in Figure S3, no changes in CG body-methylation were observed for this locus (At2g33860) in all GT lines used in this study. In the TGT-1 line, a single CG site differs in methylation from the Ws WT line, however, it is unlikely that this is the result of GT, as the TGT-1 pattern is in agreement with the published methylome of the Ws ecotype [23]. In addition, this specific site is found to be unstable among various Arabidopsis ecotypes [23]. Gene targeting events at the *CRUCIFERIN3* locus, which lacks cytosine methylation, can be identified using a seed-fluorescent assay previously described [24,25] and shown in Figure 1. We analyzed methylation in plants derived from fluorescent seeds corresponding to two GT events. The first event gave rise to a precise gene targeting allele (CRU-TGT), where the WT locus was replaced by the GT vector and with no additional non-homologous integration in the background (Figure S4). Three regions were analyzed in WT, non-targeted sibling, and the homozygous CRU-TGT line, in T2 and/or T3 generations. One region was located upstream to the *CRUCIFERIN3* promoter, beyond the region of homology between the GT cassette and the genomic target, the second, was around the *CRUCIFERIN3* translation start site, a region of homology between the GT vector and the target, and the third, was at the junction between the *CRUCIFERIN3* gene and the introduced mRFP reporter gene (yellow bars in Figure 1). For all 3 regions, no alteration in GT-related cytosine methylation was observed, indicating that the occurrence of a precise GT did not change the methylation state of a target which was formerly un-methylated (Table S3). The second GT event was not precise; as frequently happens in GT experiments, the vector invades its homologous target, but instead of replacing the target, it dissociates and integrates at an ectopic location, often capturing target sequences, while the endogenous target is left intact [7]. Such events are thus referred to as Ectopic Gene Targeting (EGT). Detailed genetic characterization of this EGT event is presented in Figure S5. The methylation patterns of the *PROHIBITIN1* gene (*PHB1*, AT4G28510) that was duplicated upon this EGT event (Figure 4) were analyzed. In contrast to the unmethylated *CRUCIFERIN3* target, the upstream gene – *PHB1* – is naturally enriched with body CG methylation. Thus, in the EGT line, we could test the methylation state of the newly duplicated *PHB1* gene as well as that of its original template locus. To this end, we separately isolated the endogenous *PHB1* and the duplicated copy for bisulfite-sequencing analysis (17). T2 and T3 generations of this EGT line were also analyzed, in order to test whether methylation of the duplicated copy is stable throughout generations. Interestingly, we found that cytosine methylation was altered following the GT-derived gene duplication at both the endogenous and the duplicated alleles, and further changed from T2 to T3 generation, in both CG and non-CG sites (Figure 4, Figure S6 and Tables S4 and S5). Increased methylation was observed at all CG dinucleotides analyzed in T2, with a most profound increase at position 18, which was unmethylated prior to targeting. In the subsequent generation (T3), methylation levels at position 18 reverted back to WT levels (0%). At the other CG sites, a decrease in methylation in the duplicated copy was observed, although not reconstructing the WT state as at position 18 (Figure 4, Figure S6 and Table S4). Although non-CG methylation is absent at the *PHB1* locus in WT, it appeared to be highly induced upon GT-derived duplication, as most (68.4%) non-CG sites were found to be 90-100% methylated in both the endogenous and the duplicated allele in the T2 generation. Interestingly, this methylation profile reverted back to the WT state already in T3, when only traces of non-CG methylation could be detected (Table S5). 

## Discussion

**Figure 5:**
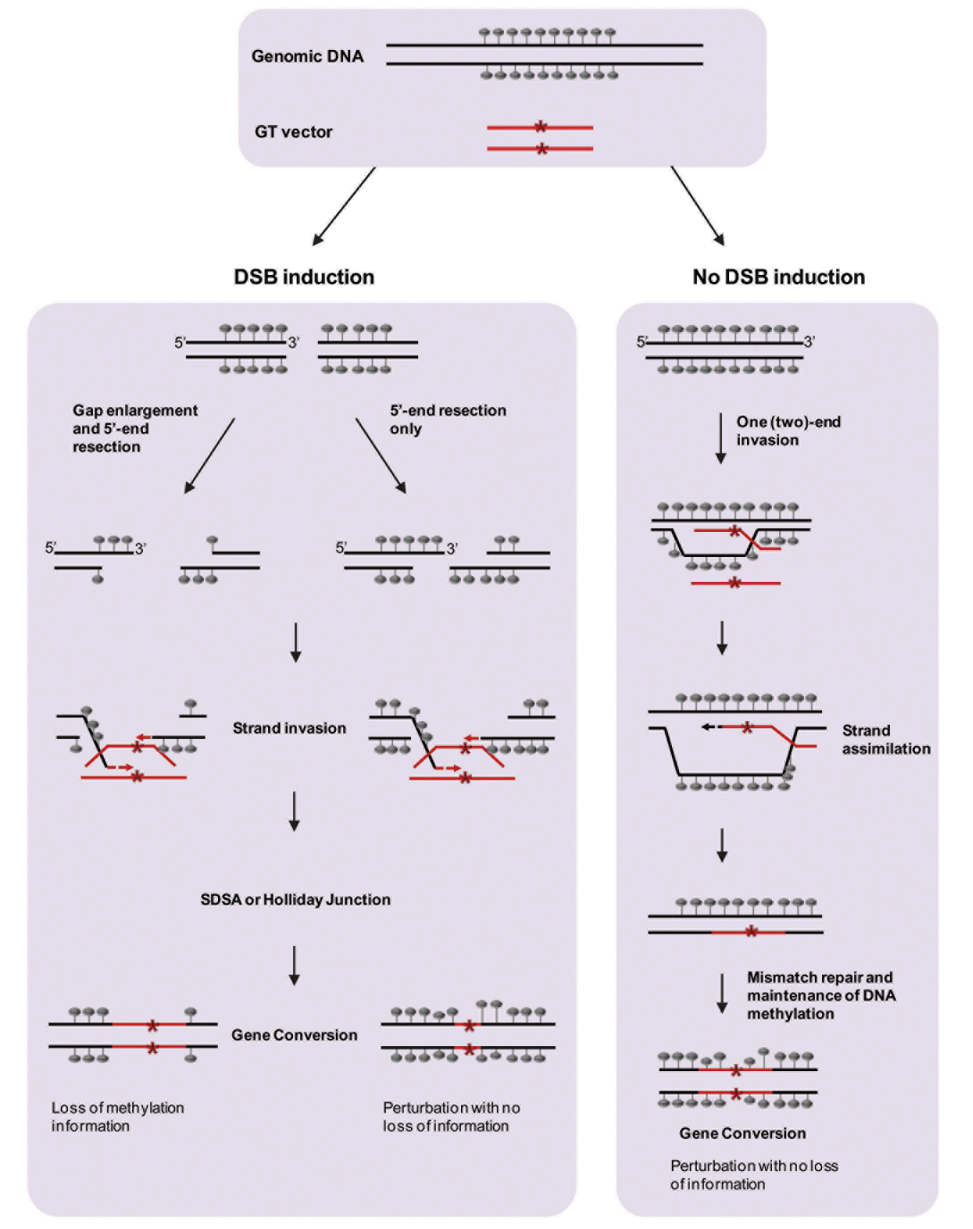
Mechanistic models for modification of methylation status of targeted alleles by GT. Methylated cytosines are indicated by grey lollipops. The GT vector is denoted by a red line, with a red asterisk illustrating the genetic perturbation introduced by the vector. If gap enlargement occurs following DSB induction (pathway on the left), the methylation template is lost and the targeted allele loses its methylation. However, if GT occurs via strand assimilation (pathway on the right) or via DSB induction but without gap enlargement (pathway in the middle), the methylation pattern of the WT can be restored upon maintenance methylation.

Taken together, our results demonstrate the possible impact of GT on the epigenetic landscape of the targeted locus. We found that several types of epigenetic alterations may occur at the targeted locus, even when the alteration is genetically precise. GT can induce complete removal of methylation, in a limited region of the target, or cause loss of stability at certain methyl-CG sites, an effect that can be further enhanced in the following generation. The changes observed suggest that the effect of GT on the methylation status of the targeted locus is not uniform or directional. The possible causes for the different effects on epigenetic alterations are not clear. One possibility is the different Arabidopsis ecotypes used for the initial GT experiments (i.e. Ws in the case of the TGT-1 line, and Col-0 for TGT-2 and 3 lines), both performed via floral-dip Agrobacterium-mediated transformation. It is known that different Arabidopsis ecotypes differ in their genome-wide methylation patterns, raising the question of whether differences in the mechanism maintaining DNA methylation exist as well, an issue that was never tested. If so, that could explain the different response to GT. It has been shown that different types of biotic stresses may cause epigenetic alterations [26], raising the possibility that the transformation event itself, carried out using bacterial infection, would trigger the observed changes in DNA methylation. To address that, we tested the methylation status of a non-target locus, where we found no changes in methylation patterns (Figure S3), suggesting that the loss of methylation at the *PPOX* locus is indeed linked and caused by the GT process. In the case of the EGT line (Figure 4, Figure S6, Table S4 and Table S5), it is likely that the changes in DNA methylation were triggered by the duplication of the *PHB1* gene, similarly to methylation changes observed following genome duplication and polyploidization, through the involvement of siRNAs and the RdDM (RNA directed DNA Methylation) pathway. Transgene silencing is yet another well studied phenomenon where the presence of repeated sequences drives hypermethylation and silencing of both copies, mediated by RdDM. It is probable that GT-mediated demethylation, as shown here in plants, can also occur in mammalian systems, due to the conserved nature of homologous recombination mechanisms. Considering the routine use of gene knockout in mammalian research this possibility should receive more attention. Indeed, earlier works have shown an alteration in allele imprinting as a result of targeted deletions or insertions [27,28], however, a detailed analysis of patterns of cytosine methylation was lacking. Note that the fate of the unmethylated DNA might differ between plants and mammals. While in mammals CG methylation can be regained de-novo, according to sequence determinants [29,30], in plants, de-novo methylation is usually of a non-CG type. Hence, loss of CG methylation in plants may result in accumulation of de-novo non-CG methylation [17,18] or, if the signal for de-novo methylation is lacking, then there is no compensation for the lost methylation and the region remains unmethylated. Epialleles have been shown to have potentially important phenotypic effects on development [31,32], in contributing to biodiversity [33] and in human disease, such as cancer [34]. In addition to its impact on gene expression and chromatin organization [13-16,35], DNA methylation was shown to be involved in essential cellular processes, such as meiotic recombination [36-38] and nucleosome positioning [39]. Therefore, the epigenetic status of a targeted locus should be taken into consideration in GT experiments, particularly when the target is methylated in the WT. In our case, we did not detect a change in expression of the *PPOX* gene, in seedlings of the TGT-1, 2, 3 lines (Figure S2). Nevertheless, since the exact role of gene body methylation is unclear, we cannot exclude an effect on another process and/or in a specific cell-type, developmental stage, or environmental condition. Targeted demethylation presents an ideal system to address the role of gene body methylation. The advances in technologies enhancing the precision and efficacy of gene targeting, such as Zinc Finger Nucleases [40], TALENs [11,41], and the more recent CRISPR [42-46], suggest that GT will become routine in several species in a near future, including plants [47-51]. In turn, the prospects of targeting genes for the sole purpose of changing their methylation status will rise. This may also provide a new tool for research or for applications in medicine and agriculture. Our results show that the changes in methylation provide new mechanistic insight into the GT process. For example, obliteration of methylation patterns during precise GT, as with the *PPOX* TGT-1 allele, provides support for the double strand break (DSB) repair model [52], whereby the DSB in the target is followed by gap enlargement and repaired via strand invasion and copying of the unmethylated vector (Figure 5). The EGT event at the *CRUCIFERIN3* locus provides support for the GT model involving vector invasion into the target and initiation of DNA synthesis, as in the synthesis-dependent strand annealing model [53]. In this model, the vector is released from the target template, and inserted into a new location, thereby generating duplication of the target sequence as well as of its original methylation imprints (Figure 5). Remarkably, this type of duplication can induce epigenetic instability in both the genetically unaltered endogenous target region and in the new duplicated locus. The case of TGT-2 and TGT-3, where original methylation was maintained at most positions, can be explained by two different models, namely the model of strand assimilation [54] or a DSB repair model, where protruding ends are generated but without gap enlargement (Figure 5). In both cases, a template is present for methylation maintenance. Interestingly, we observed methylation instability at few CG sites of the region examined. Although the mechanism of this epigenetic instability is yet to be elucidated, it is reminiscent of the genetic instability reported during mitotic gene conversion in yeast [55] where mutations resulting from errors made by DNA polymerases seemed to be affected by the local sequence context. It is possible that the observed epigenetic instability reported here results from perturbations of the maintenance methyltransferase activity. Finally, we show (in TGT-1) that DNA methylation analysis of GT events provides novel insight into the analysis of the conversion tract predicted from the DSB repair model. Namely, by using methylation polymorphism, gap enlargement can be measured at a higher resolution than previously achieved, even in the absence of DNA polymorphism. We therefore propose that DNA methylation may be exploited in future experiments to better understand the mechanism(s) of GT in higher eukaryotes.

## Supporting Information

Figure S1 **Southern blot analysis of the *PPOX*-targeted lines.** GT events at the PPOX locus were analyzed by Southern-blot hybridization using probe A [21]. Three plants of each line (different GT events or different generation) were analyzed. All plants of a given line gave the same result, therefore, only one plant per line is presented here as an example. As described previously for TGT-1 in the *Ws* background [21], using genomic DNA digested with KpnI and NcoI, the expected size for the GT allele is 2 Kb, whereas the WT allele and the T-DNA (if present) have the sizes of 11.6 Kb and 2.7 Kb, respectively. In the *Col-0* background (TGT-2 and TGT-3), expected sizes are 2 Kb, > 23 Kb and 4.2 Kb for the GT allele, WT allele and T-DNA, respectively. In this blot, we show that accurate GT was achieved and that no additional ectopic integration events occurred.
(TIF)

Figure S2 ***PPOX* transcript level in the different GT lines.** *PPOX* mRNA extracted from 7-day-old seedlings was measured by qPCR using two different primer pairs (**A**: Forward: 5'CGGGCTACGAAGGGCTAT3', Reverse: 5'ACCTCAATCGCGGTTTCA3' and **B**: Forward: 5'GCCTCAAGCCATTCCTCA3', Reverse: 5'CTTCGTAGCCCGAAGACG3'). Error bars represent variation between two biological replicates (each replicate was measured twice). PDF2 (At1g13320) and SAND (At2g28390) were used as reference genes.
(TIF)

Figure S3 **DNA methylation at the non-targeted locus At2g33860 is unchanged.** Cytosine methylation data obtained from bisulfite-sequencing of a non-target locus, in all GT lines used in this study. Each circle represents a cytosine residue, either methylated (full circle) or non-methylated (empty circle). Cytosines are color-coded by their sequence context: red for CG, blue for CHG and green for CHH (H is C, T or A). Each row represents an independent clone. (**A**) Methylation pattern in the TGT-1 line, generated in the Ws background, and a Ws-WT control. (**B**) Methylation pattern in the TGT-2 and TGT-3 lines, generated in the Col-0 background, compared to the respective Col-0 WT control.
(TIF)

Figure S4 **Genetic analysis of the CRU-TGT allele.** (**A**) Schematic illustration of a precisely targeted *CRUCIFERIN3* locus (*CRU3*). P, promoter region. mRFP, the monomeric red fluorescence protein positive marker inserted by the GT vector. HindIII and PvuII restriction sites are marked by H and P, respectively. Fragments lengths correspond to the bands shown in Southern blots in c and d. Grey rectangle, probe. Black arrows indicate positions and directions of PCR primers used to confirm precise junctions at the targeted locus. (B) PCR results for the CRU-TGT line and two independent EGT lines. (**C**) Southern blot analysis of targeted T1 plants, using PvuII digestion, showing the TGT allele (5.7 Kb). (**D**) Southern blot analysis of T2 progeny of the CRU-TGT plant, using the HindIII restriction enzyme. Analysis of only red-fluorescent seeds in T2 gave the expected 1 homozygous:2 heterozygous ratio (p (χ^2^)= 0.73).
(TIF)

Figure S5 **Genetic analysis of the EGT allele at the *CRUCIFERIN3* locus.** (**A**) Schematic illustration of the naïve *CRUCIFERIN3* locus (upper) and of the targeted locus (lower). P, promoter region. mRFP, the monomeric red fluorescence protein gene inserted by the GT vector. HindIII and PvuII restriction sites are marked by H and P, respectively. Fragment lengths correspond to the bands shown in Southern blots in b, c and d. Grey rectangles, probes. RB – the right border fragment of the GT vector that was found to be present in this EGT line. (**B**) Southern blot analysis of two EGT plants (T2 generation), using the “CRU” probe on *Hind*III-digested DNA. (**C**) Same membrane as in ‘b’ hybridized with the ‘CIFERIN’ probe, presenting the unexpected 4.2 Kb band. (**D**) Southern blot analysis of T3 progeny of the EGT line, using the *Pvu*II restriction enzyme and the ‘CIFERIN’ probe. All plants tested had the WT allele.
(TIF)

Figure S6 **CG methylation at the *PROHIBITIN1* gene.** The level of methylation is shown for each CG position in the fragment analyzed, in T2 (**A**) and T3 (**B**) generations. Red lines represent the level of methylation in a WT line, whereas the bars represent methylation of three alleles tested: the duplicated allele (black), the endogenous allele (grey) and a non-targeted sibling allele (white).
(TIF)

Table S1 **Average methylated fraction for each PPOX-targeted line.**
(DOC)

Table S2 **PPOX methylation fraction in different Col-0 WT strains.**
(DOC)

Table S3 **Average methylated fraction at the CRU3 locus in the CRU-TGT line.**
(DOC)

Table S4 **Average CG-methylated fraction at the duplicated PHB1 locus in the EGT line.**
(DOC)

Table S5 **Non-CG-methylated fraction at the duplicated PHB1 locus in the EGT line.**
(DOC)
