# Supplementary figures and images for: Epigenetic Alterations at Genomic Loci Modified by Gene Targeting in Arabidopsis thaliana
Source: PLoS One. 2013 Dec 26;8(12):e85383. doi: 10.1371/journal.pone.0085383 (PMC3873452; doi:10.1371/journal.pone.0085383)

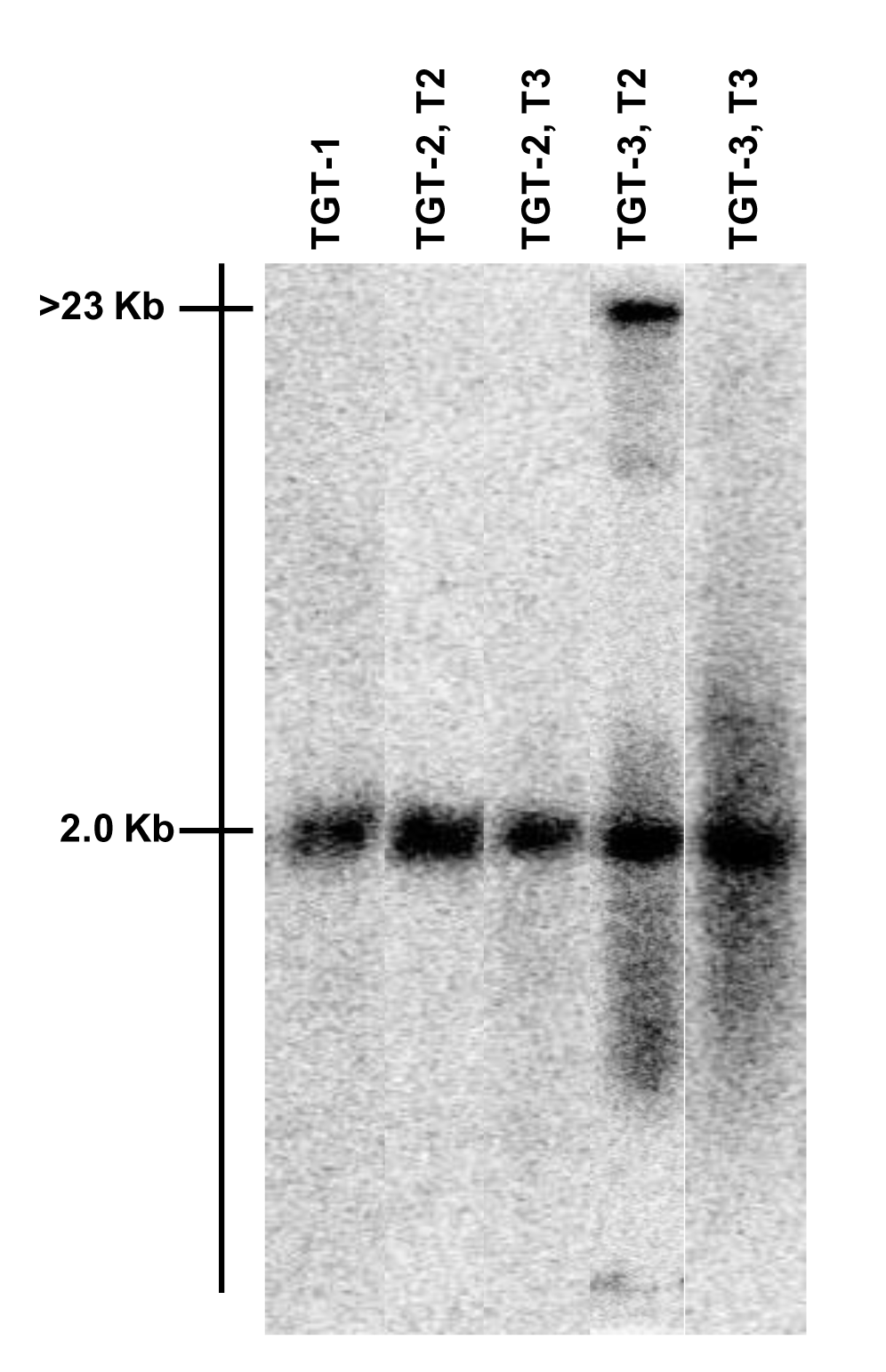

Supplement: Figure S1 — Southern blot analysis of the PPOX-targeted lines. GT events at the PPOX locus were analyzed by Southern-blot hybridization using probe A [21]. Three plants of each line (different GT events or different generation) were analyzed. All plants of a given line gave the same result, therefore, only one plant per line is presented here as an example. As described previously for TGT-1 in the Ws background [21], using genomic DNA digested with KpnI and NcoI, the expected size for the GT allele is 2 Kb, whereas the WT allele and the T-DNA (if present) have the sizes of 11.6 Kb and 2.7 Kb, respectively. In the Col-0 background (TGT-2 and TGT-3), expected sizes are 2 Kb, > 23 Kb and 4.2 Kb for the GT allele, WT allele and T-DNA, respectively. In this blot, we show that accurate GT was achieved and that no additional ectopic integration events occurred. (TIF) [file pone.0085383.s001.tif]

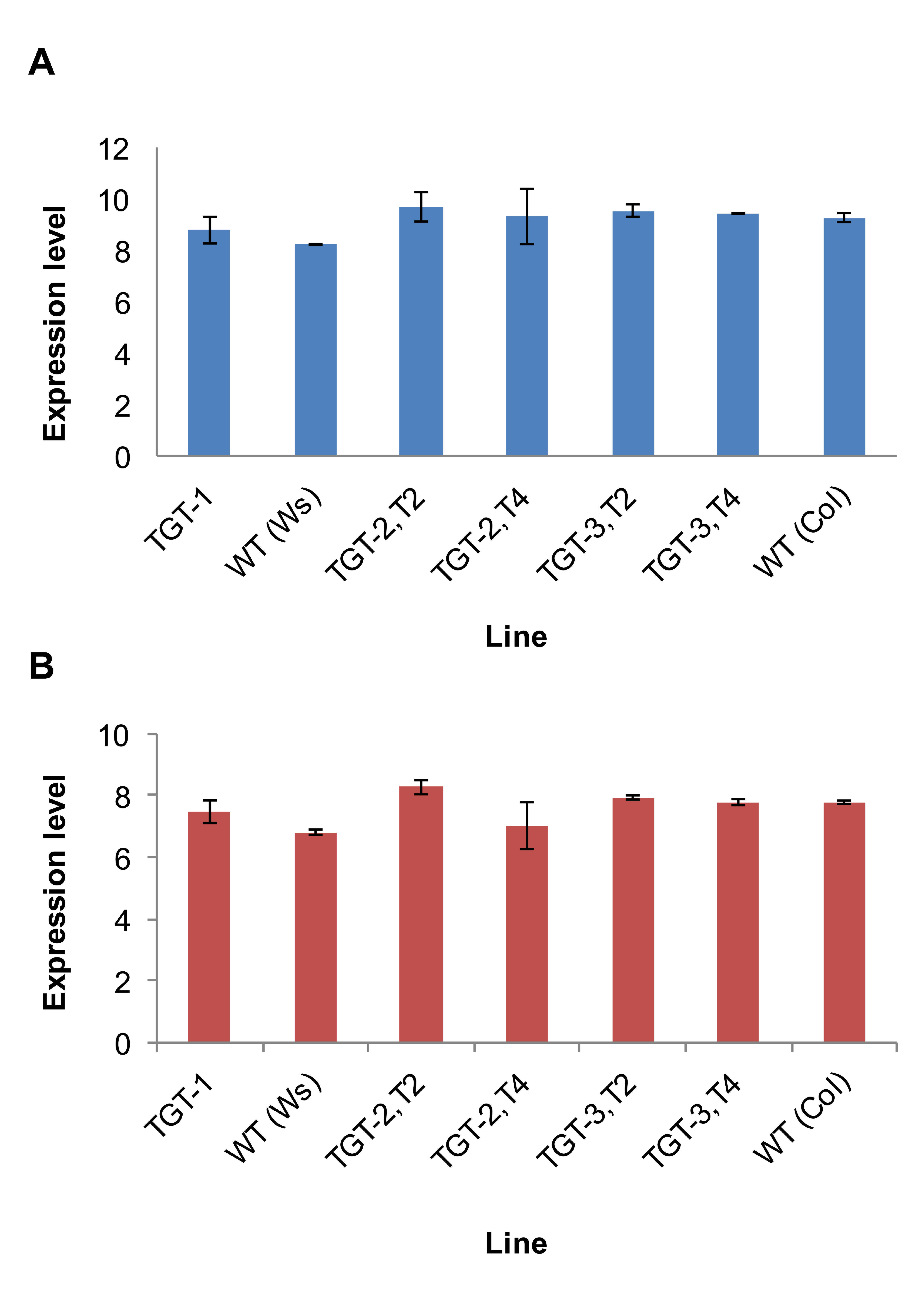

Supplement: Figure S2 — PPOX transcript level in the different GT lines. PPOX mRNA extracted from 7-day-old seedlings was measured by qPCR using two different primer pairs (A: Forward: 5'CGGGCTACGAAGGGCTAT3', Reverse: 5'ACCTCAATCGCGGTTTCA3' and B: Forward: 5'GCCTCAAGCCATTCCTCA3', Reverse: 5'CTTCGTAGCCCGAAGACG3'). Error bars represent variation between two biological replicates (each replicate was measured twice). PDF2 (At1g13320) and SAND (At2g28390) were used as reference genes. (TIF) [file pone.0085383.s002.tif]

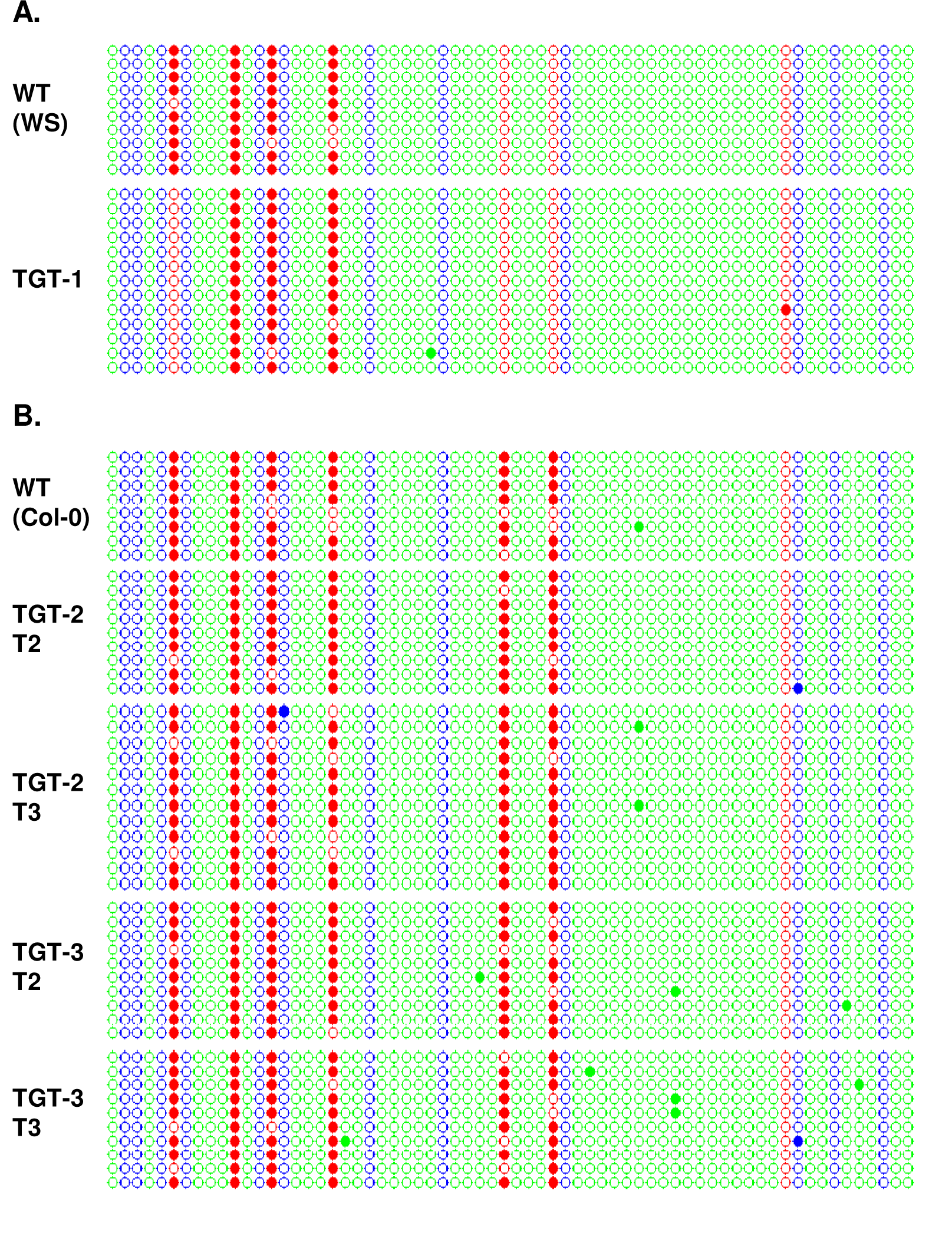

Supplement: Figure S3 — DNA methylation at the non-targeted locus At2g33860 is unchanged. Cytosine methylation data obtained from bisulfite-sequencing of a non-target locus, in all GT lines used in this study. Each circle represents a cytosine residue, either methylated (full circle) or non-methylated (empty circle). Cytosines are color-coded by their sequence context: red for CG, blue for CHG and green for CHH (H is C, T or A). Each row represents an independent clone. (A) Methylation pattern in the TGT-1 line, generated in the Ws background, and a Ws-WT control. (B) Methylation pattern in the TGT-2 and TGT-3 lines, generated in the Col-0 background, compared to the respective Col-0 WT control. (TIF) [file pone.0085383.s003.tif]

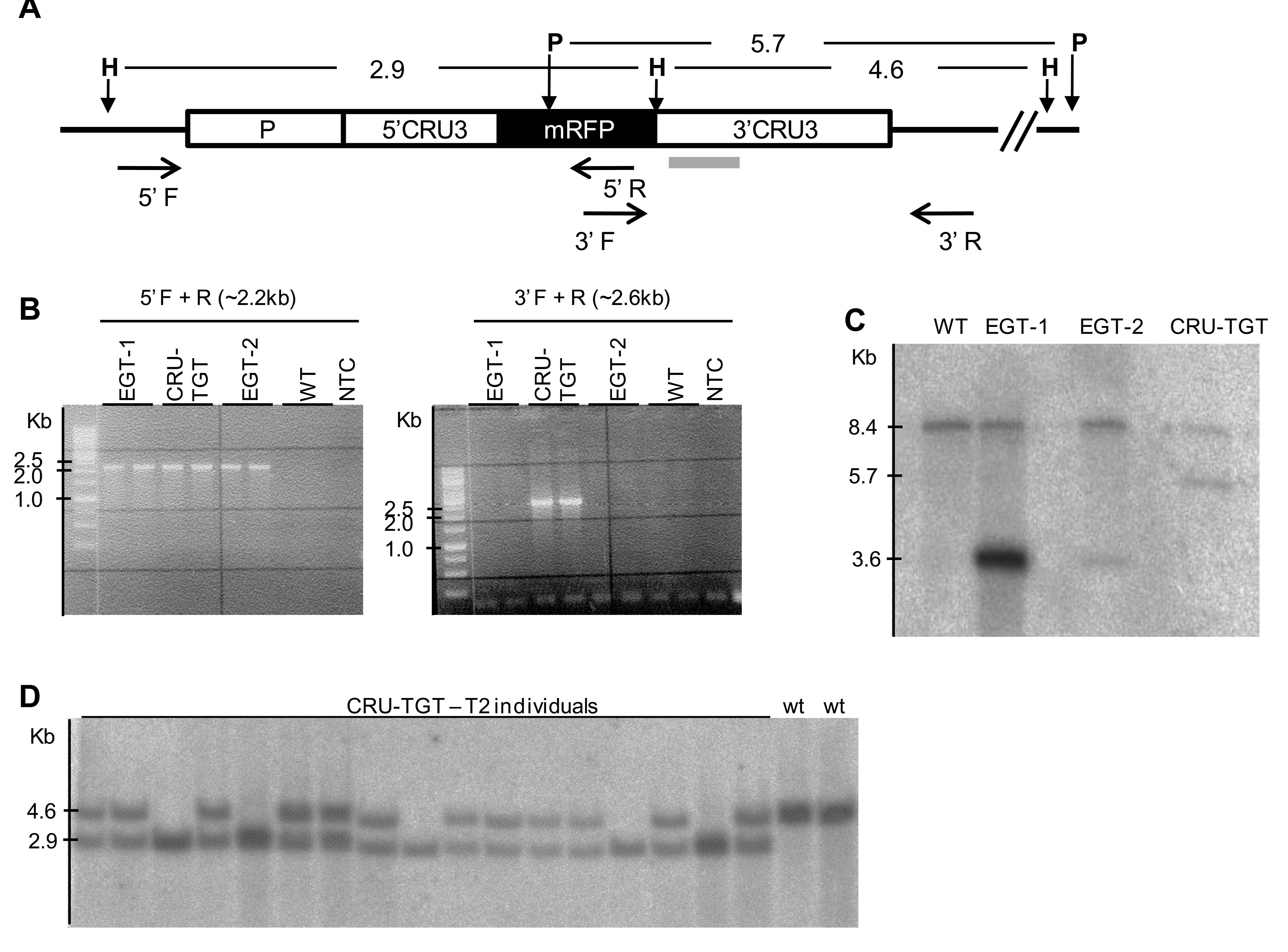

Supplement: Figure S4 — Genetic analysis of the CRU-TGT allele. (A) Schematic illustration of a precisely targeted CRUCIFERIN3 locus (CRU3). P, promoter region. mRFP, the monomeric red fluorescence protein positive marker inserted by the GT vector. HindIII and PvuII restriction sites are marked by H and P, respectively. Fragments lengths correspond to the bands shown in Southern blots in c and d. Grey rectangle, probe. Black arrows indicate positions and directions of PCR primers used to confirm precise junctions at the targeted locus. (B) PCR results for the CRU-TGT line and two independent EGT lines. (C) Southern blot analysis of targeted T1 plants, using PvuII digestion, showing the TGT allele (5.7 Kb). (D) Southern blot analysis of T2 progeny of the CRU-TGT plant, using the HindIII restriction enzyme. Analysis of only red-fluorescent seeds in T2 gave the expected 1 homozygous:2 heterozygous ratio (p (χ2)= 0.73). (TIF) [file pone.0085383.s004.tif]

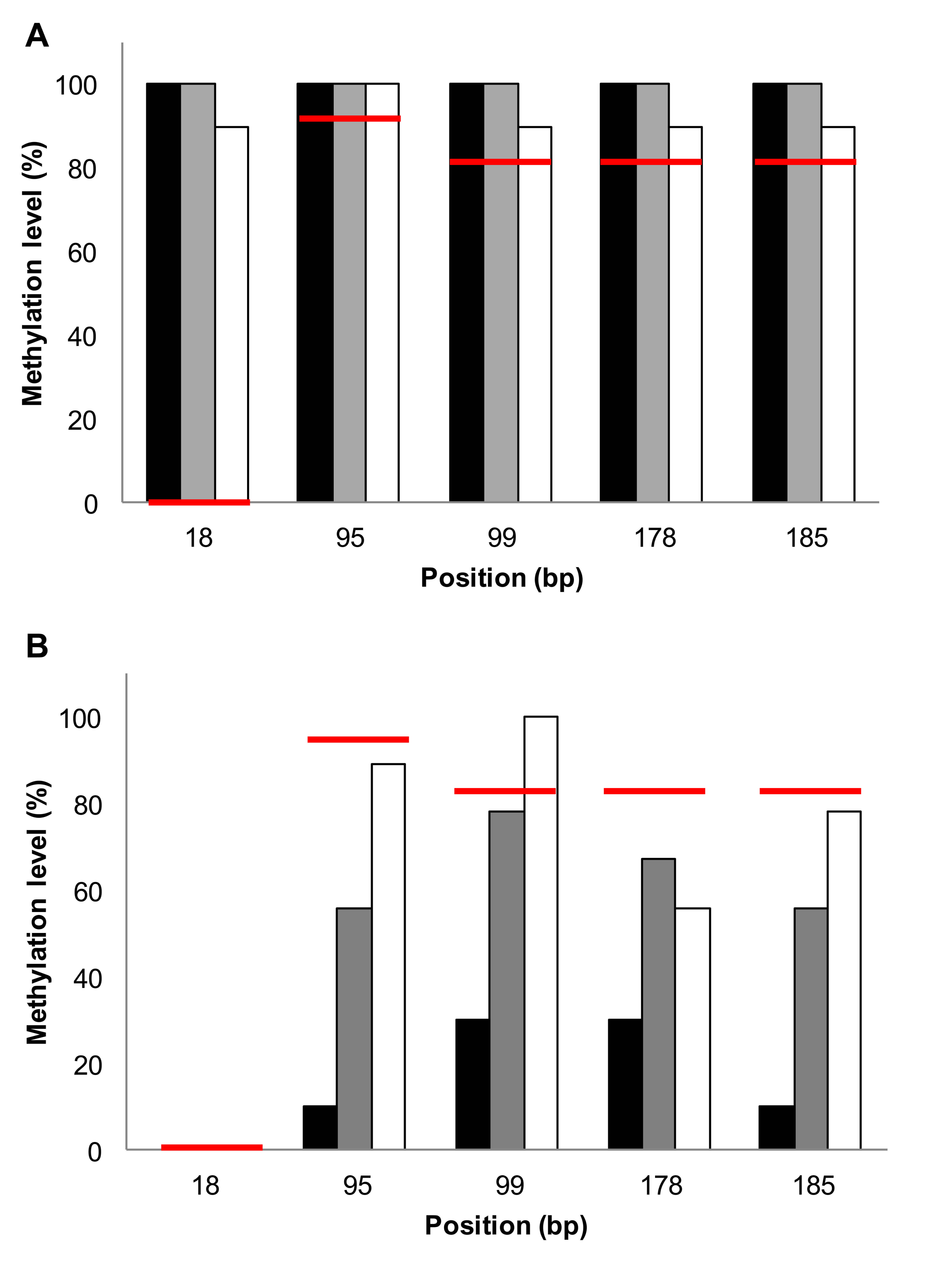

Supplement: Figure S6 — CG methylation at the PROHIBITIN1 gene. The level of methylation is shown for each CG position in the fragment analyzed, in T2 (A) and T3 (B) generations. Red lines represent the level of methylation in a WT line, whereas the bars represent methylation of three alleles tested: the duplicated allele (black), the endogenous allele (grey) and a non-targeted sibling allele (white). (TIF) [file pone.0085383.s006.tif]
