# Supplementary material for: Epigenetic Alterations at Genomic Loci Modified by Gene Targeting in Arabidopsis thaliana
Source: PLoS One. 2013 Dec 26;8(12):e85383. doi: 10.1371/journal.pone.0085383 (PMC3873452; doi:10.1371/journal.pone.0085383)
Supplement: Table S1 — Average methylated fraction for each PPOX-targeted line. (DOC) [file pone.0085383.s007.doc]

**Table S1. Average methylated fraction for each PPOX-targeted line**

| **Sample** | **no. of clones** | **CG** | **CHG** | **CHH** |
| --- | --- | --- | --- | --- |
| TGT-1 | 15 | 0.35 | 0.01 | 0.00 |
| WT (Ws) | 9 | 0.70 | 0.01 | 0.00 |
| TGT-2 (T2, heterozygous) | 24 | 0.67 | 0.08 | 0.01 |
| TGT-2 (T2, GT allele) | 13 | 0.66 | 0.09 | 0.01 |
| TGT-2 (T2, WT allele) | 11 | 0.67 | 0.08 | 0.00 |
| TGT-2 (T3) | 9 | 0.73 | 0.00 | 0.00 |
| TGT-3 (T2, heterozygous) | 21 | 0.77 | 0.13 | 0.02 |
| TGT-3 (T2, GT allele) | 11 | 0.74 | 0.20 | 0.02 |
| TGT-3 (T2, WT allele) | 10 | 0.80 | 0.05 | 0.01 |
| TGT-3 (T3) | 10 | 0.84 | 0.15 | 0.01 |
| WT (Col) | 10 | 0.68 | 0.03 | 0.01 |
