# Supplementary material for: Epigenetic Alterations at Genomic Loci Modified by Gene Targeting in Arabidopsis thaliana
Source: PLoS One. 2013 Dec 26;8(12):e85383. doi: 10.1371/journal.pone.0085383 (PMC3873452; doi:10.1371/journal.pone.0085383)
Supplement: Table S2 — PPOX methylation fraction in different Col-0 WT strains. (DOC) [file pone.0085383.s008.doc]

**Table S2. PPOX methylation fraction** in different Col-0 WT strains

| **Position** | **Site type** | **WT-1** | **WT-2** | **WT-3** | **WT-4** | **WT-5** | **WT-6** | **Average** |
| --- | --- | --- | --- | --- | --- | --- | --- | --- |
| 5 | CHH | 0.00 | 0.00 | 0.00 | 0.00 | 0.13 | 0.00 | 0.02 |
| 11 | CHH | 0.00 | 0.00 | 0.00 | 0.00 | 0.13 | 0.00 | 0.02 |
| 14 | CHH | 0.00 | 0.00 | 0.00 | 0.00 | 0.00 | 0.00 | 0.00 |
| 15 | CHH | 0.00 | 0.00 | 0.13 | 0.00 | 0.00 | 0.00 | 0.02 |
| **17** | **CG** | **0.71** | **0.63** | **0.90** | **1.00** | **0.89** | **0.93** | **0.84** |
| 19 | CHH | 0.00 | 0.00 | 0.00 | 0.00 | 0.00 | 0.00 | 0.00 |
| **21** | **CG** | **0.71** | **1.00** | **0.90** | **1.00** | **1.00** | **0.86** | **0.91** |
| 23 | CHH | 0.00 | 0.00 | 0.00 | 0.00 | 0.00 | 0.00 | 0.00 |
| 36 | CHH | 0.00 | 0.00 | 0.00 | 0.00 | 0.00 | 0.00 | 0.00 |
| 50 | CHH | 0.00 | 0.13 | 0.00 | 0.00 | 0.00 | 0.00 | 0.02 |
| **64** | **CG** | **0.00** | **0.25** | **0.00** | **0.00** | **0.00** | **0.00** | **0.04** |
| 67 | CHH | 0.00 | 0.13 | 0.00 | 0.00 | 0.00 | 0.00 | 0.02 |
| 76 | CHH | 0.00 | 0.00 | 0.00 | 0.00 | 0.00 | 0.00 | 0.00 |
| 85 | CHH | 0.00 | 0.00 | 0.00 | 0.00 | 0.00 | 0.00 | 0.00 |
| 93 | CHH | 0.00 | 0.00 | 0.00 | 0.00 | 0.00 | 0.00 | 0.00 |
| 96 | CHH | 0.00 | 0.00 | 0.00 | 0.00 | 0.00 | 0.00 | 0.00 |
| 100 | CHG | 0.00 | 0.13 | 0.00 | 0.00 | 0.11 | 0.00 | 0.04 |
| 106 | CHH | 0.00 | 0.00 | 0.00 | 0.00 | 0.11 | 0.00 | 0.02 |
| **108** | **CG** | **1.00** | **0.50** | **0.80** | **1.00** | **1.00** | **1.00** | **0.88** |
| 115 | CHH | 0.00 | 0.00 | 0.00 | 0.00 | 0.00 | 0.00 | 0.00 |
| 118 | CHG | 0.00 | 0.13 | 0.00 | 0.00 | 0.00 | 0.00 | 0.02 |
| 124 | CHH | 0.00 | 0.13 | 0.00 | 0.00 | 0.00 | 0.00 | 0.02 |
| 128 | CHH | 0.00 | 0.00 | 0.00 | 0.00 | 0.00 | 0.00 | 0.00 |
| 131 | CHG | 0.00 | 0.00 | 0.00 | 0.00 | 0.00 | 0.00 | 0.00 |
| 134 | CHH | 0.00 | 0.13 | 0.00 | 0.00 | 0.11 | 0.00 | 0.04 |
| 136 | CHH | 0.00 | 0.00 | 0.00 | 0.00 | 0.00 | 0.07 | 0.01 |
| 137 | CHH | 0.00 | 0.00 | 0.00 | 0.00 | 0.00 | 0.00 | 0.00 |
| 139 | CHH | 0.00 | 0.00 | 0.00 | 0.00 | 0.11 | 0.00 | 0.02 |
| 141 | CHH | 0.00 | 0.00 | 0.00 | 0.00 | 0.00 | 0.00 | 0.00 |
| 143 | CHH | 0.00 | 0.13 | 0.00 | 0.00 | 0.00 | 0.00 | 0.02 |
| 147 | CHH | 0.00 | 0.00 | 0.00 | 0.00 | 0.00 | 0.00 | 0.00 |
| 148 | CHH | 0.00 | 0.00 | 0.00 | 0.00 | 0.11 | 0.00 | 0.02 |
| **153** | **CG** | **0.86** | **0.88** | **0.80** | **0.88** | **1.00** | **0.86** | **0.88** |
| **155** | **CG** | **0.57** | **0.13** | **0.30** | **1.00** | **1.00** | **0.93** | **0.65** |
| 157 | CHH | 0.00 | 0.00 | 0.00 | 0.00 | 0.00 | 0.00 | 0.00 |
| 159 | CHG | 0.00 | 0.25 | 0.00 | 0.00 | 0.11 | 0.00 | 0.06 |
| **160** | **CG** | **1.00** | **1.00** | **0.90** | **1.00** | **1.00** | **0.93** | **0.97** |
| 162 | CHH | 0.00 | 0.13 | 0.00 | 0.00 | 0.00 | 0.00 | 0.02 |
| 163 | CHG | 0.00 | 0.00 | 0.00 | 0.00 | 0.11 | 0.00 | 0.02 |
| **164** | **CG** | **1.00** | **0.63** | **1.00** | **0.88** | **0.89** | **0.93** | **0.89** |

**Table S2. PPOX methylation fraction in different Col-0 WT strains – continued**

| **Position** | **Site type** | **WT-1** | **WT-2** | **WT-3** | **WT-4** | **WT-5** | **WT-6** | **Average** |
| --- | --- | --- | --- | --- | --- | --- | --- | --- |
| 177 | CHG | 0.00 | 0.13 | 0.00 | 0.00 | 0.00 | 0.00 | 0.02 |
| 185 | CHH | 0.00 | 0.00 | 0.00 | 0.00 | 0.00 | 0.00 | 0.00 |
| 188 | CHH | 0.00 | 0.13 | 0.00 | 0.00 | 0.00 | 0.00 | 0.02 |
| **194** | **CG** | **0.00** | **0.13** | **0.00** | **0.00** | **0.00** | **0.29** | **0.07** |
| 199 | CHH | 0.00 | 0.13 | 0.00 | 0.00 | 0.00 | 0.00 | 0.02 |
| 202 | CHH | 0.00 | 0.00 | 0.00 | 0.00 | 0.00 | 0.00 | 0.00 |
| 206 | CHH | 0.00 | 0.00 | 0.00 | 0.00 | 0.22 | 0.00 | 0.04 |
| 208 | CHG | 0.00 | 0.00 | 0.10 | 0.00 | 0.00 | 0.00 | 0.02 |
| **209** | **CG** | **0.86** | **1.00** | **0.70** | **0.75** | **0.67** | **0.93** | **0.82** |
| 216 | CHG | 0.00 | 0.13 | 0.00 | 0.00 | 0.00 | 0.00 | 0.02 |
| 220 | CHH | 0.00 | 0.13 | 0.00 | 0.00 | 0.22 | 0.00 | 0.06 |
| 221 | CHH | 0.00 | 0.13 | 0.00 | 0.00 | 0.11 | 0.00 | 0.04 |
| 233 | CHG | 0.00 | 0.13 | 0.00 | 0.00 | 0.11 | 0.00 | 0.04 |
| 236 | CHH | 0.00 | 0.13 | 0.00 | 0.00 | 0.11 | 0.00 | 0.04 |
| 240 | CHH | 0.00 | 0.00 | 0.00 | 0.00 | 0.22 | 0.00 | 0.04 |
| 242 | CHH | 0.00 | 0.00 | 0.00 | 0.00 | 0.00 | 0.00 | 0.00 |
| 249 | CHH | 0.00 | 0.00 | 0.00 | 0.00 | 0.11 | 0.00 | 0.02 |
| 251 | CHH | 0.00 | 0.13 | 0.00 | 0.00 | 0.00 | 0.00 | 0.02 |
| 254 | CHH | 0.00 | 0.00 | 0.00 | 0.00 | 0.00 | 0.00 | 0.00 |
| 261 | CHH | 0.00 | 0.00 | 0.00 | 0.00 | 0.00 | 0.00 | 0.00 |
| 264 | CHH | 0.00 | 0.13 | 0.00 | 0.00 | 0.00 | 0.00 | 0.02 |
| 265 | CHH | 0.00 | 0.13 | 0.00 | 0.00 | 0.00 | 0.00 | 0.02 |
| 273 | CHH | 0.00 | 0.00 | 0.00 | 0.00 | 0.00 | 0.00 | 0.00 |
| 274 | CHH | 0.00 | 0.00 | 0.00 | 0.00 | 0.00 | 0.00 | 0.00 |
| 281 | CHG | 0.00 | 0.13 | 0.00 | 0.00 | 0.00 | 0.00 | 0.02 |
| 292 | CHH | 0.00 | 0.00 | 0.10 | 0.00 | 0.00 | 0.00 | 0.02 |
| 295 | CHH | 0.00 | 0.00 | 0.00 | 0.00 | 0.00 | 0.00 | 0.00 |
| 297 | CHH | 0.00 | 0.00 | 0.10 | 0.00 | 0.00 | 0.00 | 0.02 |
| 299 | CHH | 0.00 | 0.13 | 0.10 | 0.00 | 0.00 | 0.00 | 0.04 |
| 301 | CHH | 0.00 | 0.00 | 0.10 | 0.00 | 0.00 | 0.00 | 0.02 |
| 303 | CHH | 0.00 | 0.13 | 0.10 | 0.00 | 0.00 | 0.00 | 0.04 |
| 305 | CHH | 0.00 | 0.00 | 0.10 | 0.00 | 0.11 | 0.00 | 0.04 |
| 319 | CHG | 0.00 | 0.00 | 0.10 | 0.00 | 0.00 | 0.00 | 0.02 |
| **320** | **CG** | **0.14** | **0.13** | **0.10** | **0.88** | **1.00** | **1.00** | **0.54** |
| 322 | CHG | 0.00 | 0.00 | 0.10 | 0.14 | 0.00 | 0.00 | 0.04 |
| 326 | CHG | 0.00 | 0.00 | 0.00 | 0.00 | 0.11 | 0.00 | 0.02 |
