# Supplementary material for: Epigenetic Alterations at Genomic Loci Modified by Gene Targeting in Arabidopsis thaliana
Source: PLoS One. 2013 Dec 26;8(12):e85383. doi: 10.1371/journal.pone.0085383 (PMC3873452; doi:10.1371/journal.pone.0085383)
Supplement: Table S3 — Average methylated fraction at the CRU3 locus in the CRU-TGT line. (DOC) [file pone.0085383.s009.doc]

**Table S3. Average methylated fraction at the CRU3 locus in the CRU-TGT line**

| **Fragment** | **Sample** | **no. of clones** | **CG** | **CHG** | **CHH** |
| --- | --- | --- | --- | --- | --- |
| CRU promoter | T2_homozygous | 7 | 0.00 | 0.00 | 0.00 |
| CRU promoter | T3_homozygous | 5 | 0.00 | 0.00 | 0.00 |
| CRU promoter | N | 8 | 0.00 | 0.00 | 0.00 |
| CRU promoter | WT | 8 | 0.00 | 0.00 | 0.00 |
| GT junction | T2_homozygous | 10 | 0.00 | 0.00 | 0.00 |
| GT junction | N | 10 | 0.00 | 0.00 | 0.00 |
| GT junction | WT | 10 | 0.01 | 0.00 | 0.00 |
| mRFP insertion | T3_homozygous | 10 | 0.00 | 0.00 | 0.00 |

N, A non-fluorescent segregating line
