# Supplementary material for: Epigenetic Alterations at Genomic Loci Modified by Gene Targeting in Arabidopsis thaliana
Source: PLoS One. 2013 Dec 26;8(12):e85383. doi: 10.1371/journal.pone.0085383 (PMC3873452; doi:10.1371/journal.pone.0085383)
Supplement: Table S4 — Average CG-methylated fraction at the duplicated PHB1 locus in the EGT line. (DOC) [file pone.0085383.s010.doc]

**Table S4. Average CG-methylated fraction at the duplicated PHB1 locus in the EGT line**

| **CG position (bp)** | **T2** | | | **T3** | | | **WT** |
| --- | --- | --- | --- | --- | --- | --- | --- |
|  | **DA** | **WA** | **NT** | **DA** | **WA** | **NT** |  |
| 18 | 1.00 | 1.00 | 0.90 | 0.00 | 0.00 | 0.00 | 0.00 |
| 95 | 1.00 | 1.00 | 1.00 | 0.10 | 0.56 | 0.89 | 0.90 |
| 99 | 1.00 | 1.00 | 0.90 | 0.30 | 0.78 | 1.00 | 0.80 |
| 178 | 1.00 | 1.00 | 0.90 | 0.30 | 0.67 | 0.56 | 0.80 |
| 185 | 1.00 | 1.00 | 0.90 | 0.10 | 0.56 | 0.78 | 0.80 |
| **Average** | 1.00 | 1.00 | 0.92 | 0.16 | 0.51 | 0.64 | 0.66 |
| **no. of clones** | 10 | 10 | 10 | 10 | 9 | 9 | 10 |

DA – duplicated allele, WA – WT (endogenous) allele, NT – non-targeted allele (WT sibling of the EGT line)
