# Supplementary material for: Epigenetic Alterations at Genomic Loci Modified by Gene Targeting in Arabidopsis thaliana
Source: PLoS One. 2013 Dec 26;8(12):e85383. doi: 10.1371/journal.pone.0085383 (PMC3873452; doi:10.1371/journal.pone.0085383)
Supplement: Table S5 — Non-CG-methylated fraction at the duplicated PHB1 locus in the EGT line. (DOC) [file pone.0085383.s011.doc]

**Table S5. Non-CG-methylated fraction at the duplicated PHB1 locus in the EGT line**

| position (bp( | sequence context | methylation in wt | Fraction of methylation in the EGT line | | | | | |
| --- | --- | --- | --- | --- | --- | --- | --- | --- |
|  |  |  | T2 DA | T2 WA | T2 NT | T3 DA | T3 WA | T3 NT |
| 2 | CHG | 0.00 | 1.00 | 1.00 | 0.90 | 0.00 | 0.00 | 0.00 |
| 7 | CHG | 0.00 | 1.00 | 1.00 | 1.00 | 0.00 | 0.11 | 0.00 |
| 10 | CHH | 0.00 | 1.00 | 1.00 | 1.00 | 0.00 | 0.00 | 0.00 |
| 11 | CHH | 0.00 | 1.00 | 1.00 | 1.00 | 0.00 | 0.00 | 0.00 |
| 28 | CHH | 0.00 | 1.00 | 1.00 | 0.90 | 0.00 | 0.00 | 0.00 |
| 29 | CHH | 0.00 | 1.00 | 1.00 | 0.90 | 0.00 | 0.00 | 0.00 |
| 30 | CHH | 0.00 | 1.00 | 1.00 | 0.50 | 0.00 | 0.00 | 0.00 |
| 35 | CHH | 0.00 | 1.00 | 1.00 | 0.50 | 0.00 | 0.00 | 0.00 |
| 36 | CHH | 0.00 | 1.00 | 1.00 | 0.00 | 0.00 | 0.00 | 0.00 |
| 37 | CHH | 0.00 | 1.00 | 1.00 | 0.50 | 0.00 | 0.00 | 0.00 |
| 44 | CHH | 0.00 | 1.00 | 1.00 | 0.90 | 0.00 | 0.00 | 0.00 |
| 46 | CHH | 0.00 | 1.00 | 1.00 | 0.90 | 0.00 | 0.00 | 0.00 |
| 47 | CHG | 0.00 | 1.00 | 1.00 | 0.50 | 0.00 | 0.00 | 0.00 |
| 62 | CHH | 0.00 | 1.00 | 1.00 | 1.00 | 0.00 | 0.00 | 0.00 |
| 63 | CHH | 0.00 | 1.00 | 1.00 | 1.00 | 0.00 | 0.00 | 0.00 |
| 65 | CHH | 0.00 | 1.00 | 1.00 | 1.00 | 0.00 | 0.00 | 0.00 |
| 72 | CHH | 0.00 | 1.00 | 1.00 | 0.90 | 0.00 | 0.00 | 0.00 |
| 80 | CHH | 0.00 | 1.00 | 1.00 | 0.90 | 0.00 | 0.00 | 0.00 |
| 102 | CHH | 0.00 | 1.00 | 1.00 | 0.40 | 0.00 | 0.00 | 0.00 |
| 105 | CHH | 0.00 | 1.00 | 1.00 | 1.00 | 0.00 | 0.00 | 0.00 |
| 107 | CHH | 0.00 | 1.00 | 1.00 | 1.00 | 0.00 | 0.11 | 0.00 |
| 111 | CHH | 0.00 | 0.90 | 1.00 | 0.10 | 0.00 | 0.00 | 0.00 |
| 113 | CHH | 0.00 | 1.00 | 1.00 | 1.00 | 0.00 | 0.00 | 0.11 |
| 120 | CHH | 0.00 | 1.00 | 1.00 | 0.60 | 0.00 | 0.00 | 0.00 |
| 122 | CHH | 0.00 | 1.00 | 1.00 | 0.60 | 0.00 | 0.00 | 0.00 |
| 127 | CHH | 0.00 | 0.00 | 1.00 | 0.40 | 0.00 | 0.00 | 0.00 |
| 135 | CHG | 0.00 | 0.00 | 1.00 | 0.90 | 0.00 | 0.00 | 0.00 |
| 157 | CHG | 0.00 | 0.00 | 1.00 | 0.90 | 0.00 | 0.00 | 0.00 |
| 164 | CHH | 0.00 | 0.00 | 1.00 | 0.90 | 0.00 | 0.00 | 0.00 |
| 165 | CHH | 0.00 | 0.00 | 1.00 | 0.90 | 0.00 | 0.00 | 0.00 |
| 168 | CHG | 0.00 | 0.00 | 1.00 | 0.90 | 0.00 | 0.00 | 0.00 |
| 176 | CHH | 0.00 | 0.00 | 1.00 | 0.90 | 0.00 | 0.00 | 0.00 |
| 181 | CHH | 0.00 | 0.00 | 1.00 | 0.00 | 0.00 | 0.00 | 0.00 |
| 184 | CHG | 0.00 | 0.00 | 1.00 | 0.90 | 0.00 | 0.00 | 0.00 |
| 193 | CHH | 0.00 | 0.00 | 1.00 | 0.90 | 0.00 | 0.00 | 0.00 |
| 195 | CHH | 0.00 | 1.00 | 1.00 | 0.90 | 0.00 | 0.00 | 0.00 |
| 197 | CHH | 0.00 | 0.00 | 1.00 | 0.50 | 0.00 | 0.00 | 0.00 |
| 200 | CHH | 0.00 | 0.00 | 1.00 | 0.90 | 0.00 | 0.00 | 0.00 |
| Average | CHG | 0.00 | 0.43 | 1.00 | 0.86 | 0.00 | 0.02 | 0.00 |
|  | CHH | 0.00 | 0.74 | 1.00 | 0.74 | 0.00 | 0.00 | 0.00 |

DA – duplicated allele, WA – WT (endogenous) allele, NT – non-targeted allele (WT sibling of the EGT line)
